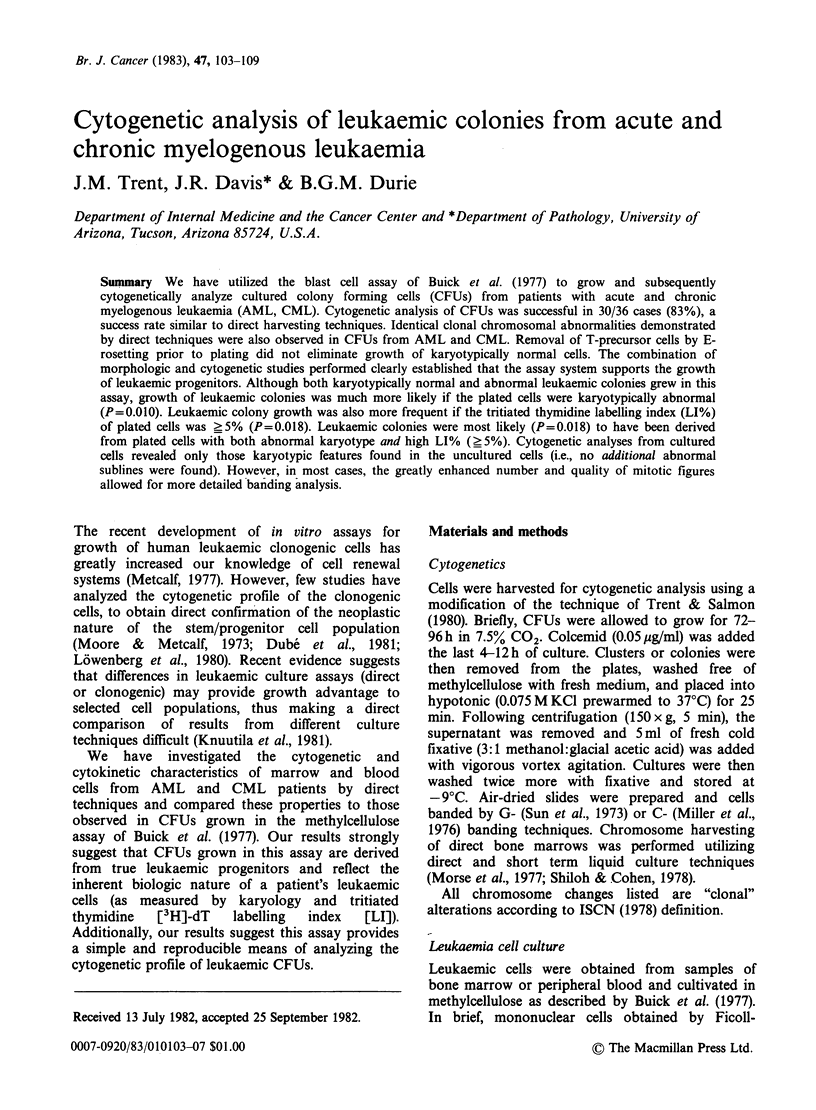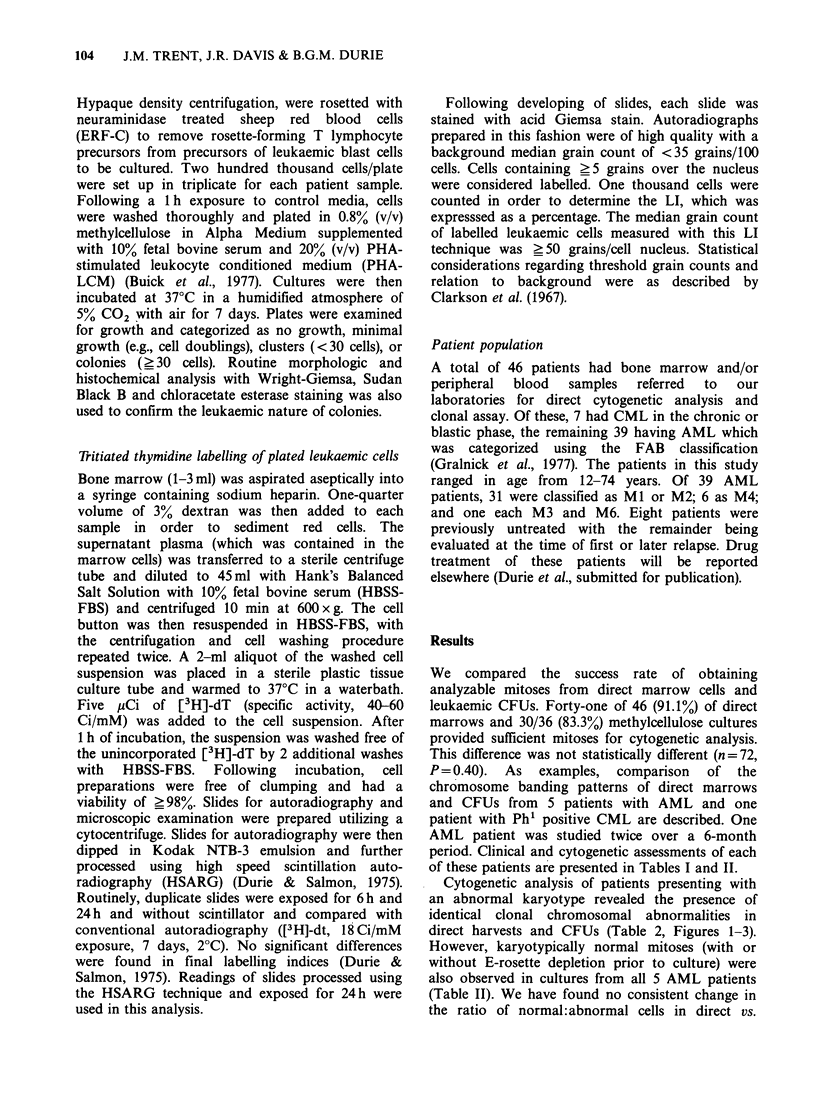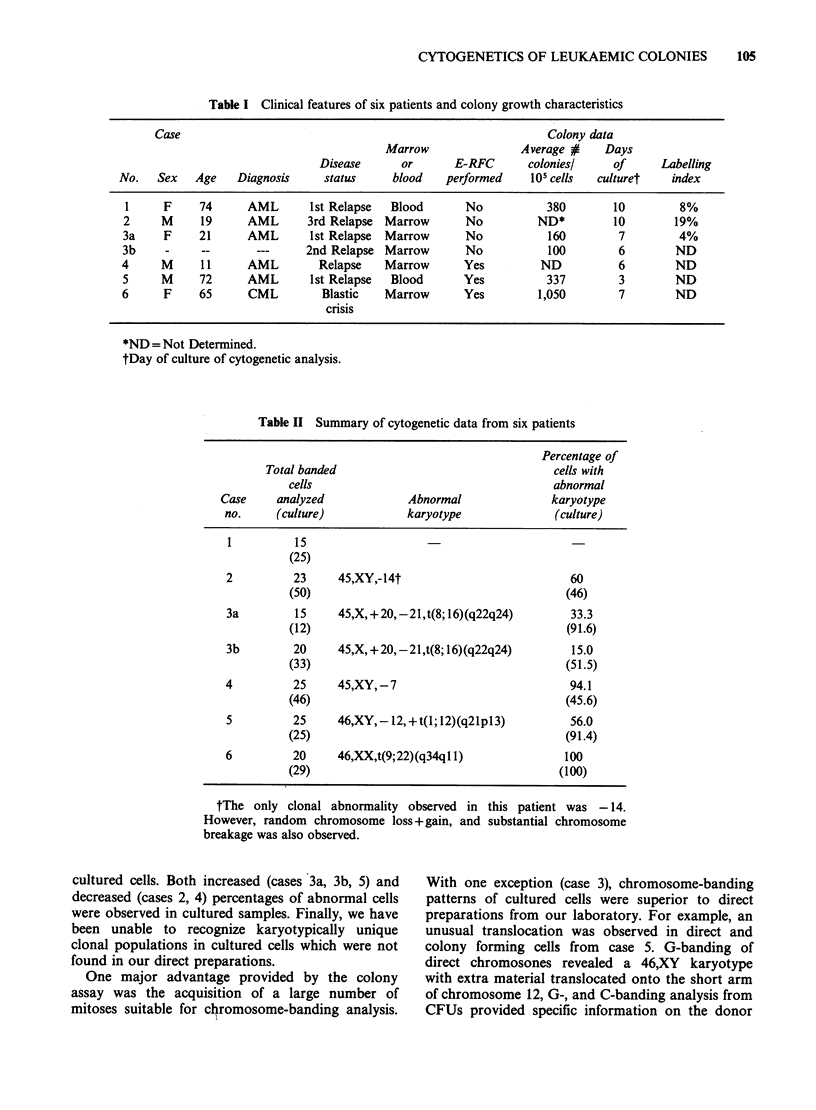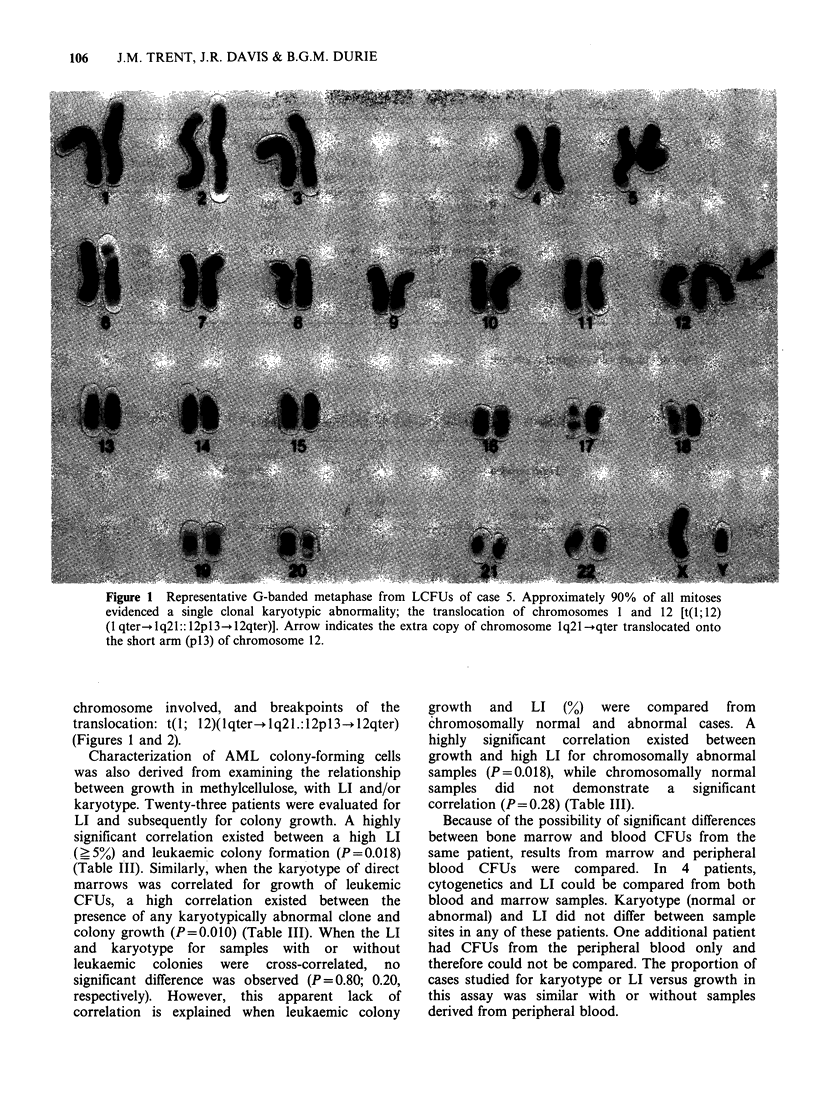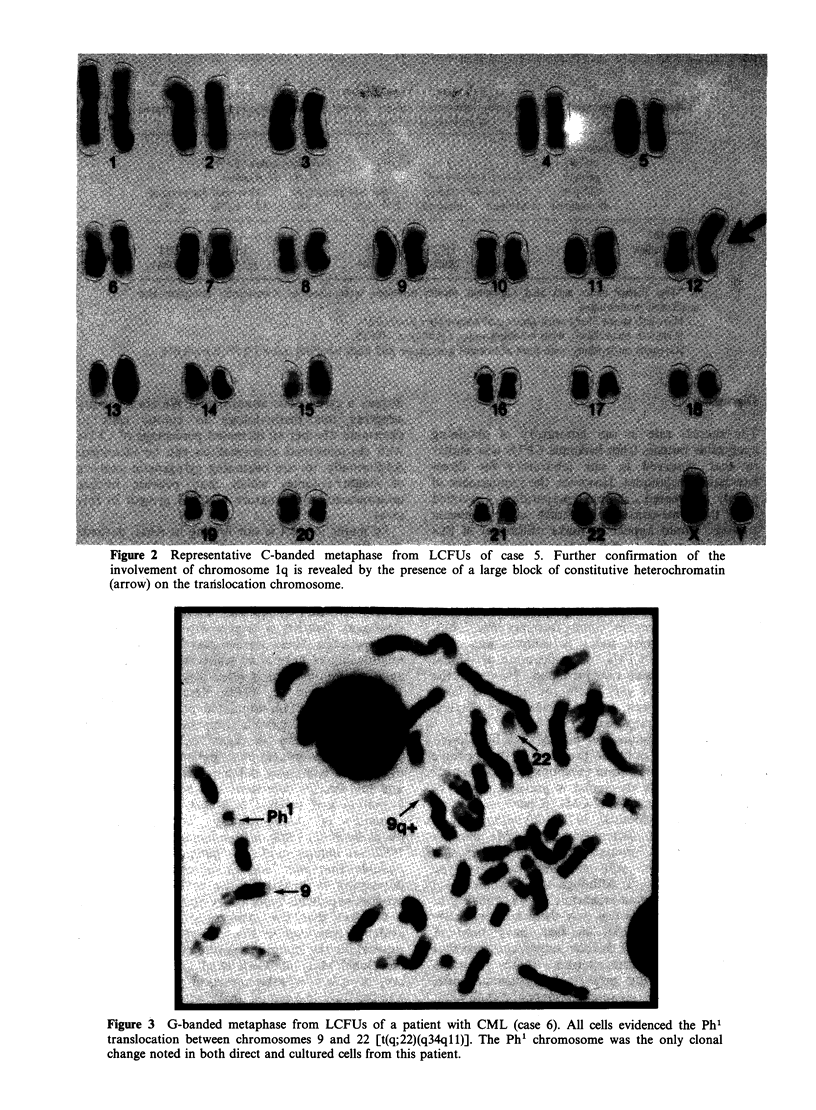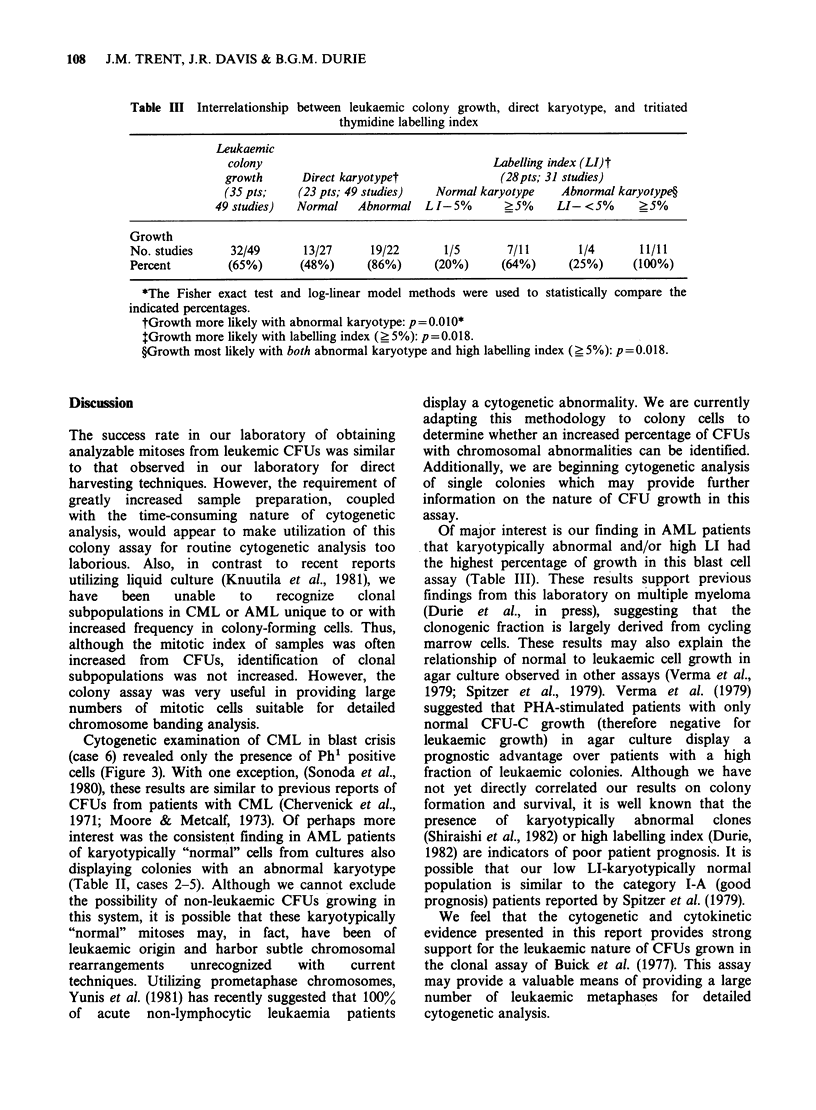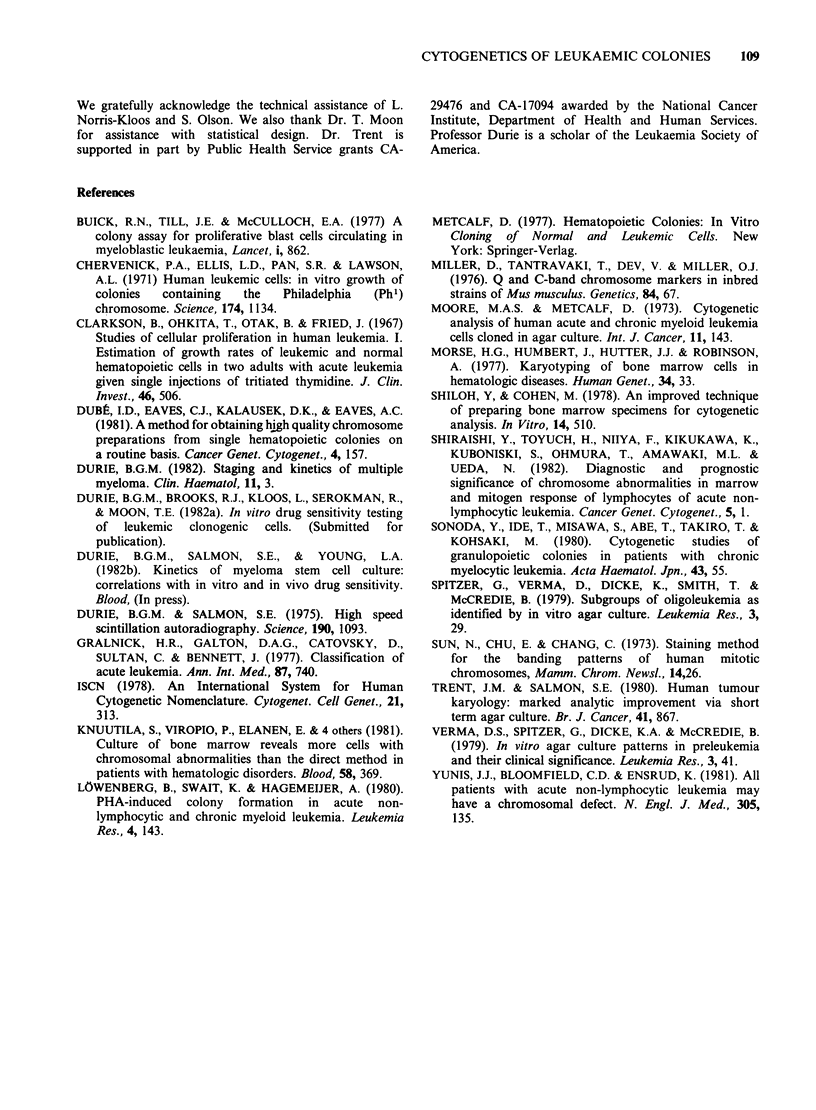# Cytogenetic analysis of leukaemic colonies from acute and chronic myelogenous leukaemia.

**DOI:** 10.1038/bjc.1983.12

**Published:** 1983-01

**Authors:** J. M. Trent, J. R. Davis, B. G. Durie

## Abstract

**Images:**


*Br. J. Cancer* (1983), **47**, 103–109

### Cytogenetic analysis of leukaemic colonies from acute and chronic myelogenous leukaemia

J.M. Trent, J.R. Davis* & B.G.M. Durie

*Department of Internal Medicine and the Cancer Center and *Department of Pathology, University of Arizona, Tucson, Arizona 85724, U.S.A.*

**Summary** We have utilized the blast cell assay of Buick *et al.* (1977) to grow and subsequently cytogenetically analyze cultured colony forming cells (CFUs) from patients with acute and chronic myelogenous leukaemia (AML, CML). Cytogenetic analysis of CFUs was successful in 30/36 cases (83%), a success rate similar to direct harvesting techniques. Identical clonal chromosomal abnormalities demonstrated by direct techniques were also observed in CFUs from AML and CML. Removal of T-precursor cells by E-rosetting prior to plating did not eliminate growth of karyotypically normal cells. The combination of morphologic and cytogenetic studies performed clearly established that the assay system supports the growth of leukaemic progenitors. Although both karyotypically normal and abnormal leukaemic colonies grew in this assay, growth of leukaemic colonies was much more likely if the plated cells were karyotypically abnormal ($P=0.010$). Leukaemic colony growth was also more frequent if the tritiated thymidine labelling index (LI%) of plated cells was $\geqq 5\%$ ($P=0.018$). Leukaemic colonies were most likely ($P=0.018$) to have been derived from plated cells with both abnormal karyotype *and* high LI% ($\geqq 5\%$). Cytogenetic analyses from cultured cells revealed only those karyotypic features found in the uncultured cells (i.e., no *additional* abnormal sublines were found). However, in most cases, the greatly enhanced number and quality of mitotic figures allowed for more detailed banding analysis.

The recent development of *in vitro* assays for growth of human leukaemic clonogenic cells has greatly increased our knowledge of cell renewal systems (Metcalf, 1977). However, few studies have analyzed the cytogenetic profile of the clonogenic cells, to obtain direct confirmation of the neoplastic nature of the stem/progenitor cell population (Moore & Metcalf, 1973; Dubé *et al.*, 1981; Löwenberg *et al.*, 1980). Recent evidence suggests that differences in leukaemic culture assays (direct or clonogenic) may provide growth advantage to selected cell populations, thus making a direct comparison of results from different culture techniques difficult (Knuutila *et al.*, 1981).

We have investigated the cytogenetic and cytokinetic characteristics of marrow and blood cells from AML and CML patients by direct techniques and compared these properties to those observed in CFUs grown in the methylcellulose assay of Buick *et al.* (1977). Our results strongly suggest that CFUs grown in this assay are derived from true leukaemic progenitors and reflect the inherent biologic nature of a patient's leukaemic cells (as measured by karyology and tritiated thymidine [$^3$H]-dT labelling index [LI]). Additionally, our results suggest this assay provides a simple and reproducible means of analyzing the cytogenetic profile of leukaemic CFUs.

Received 13 July 1982, accepted 25 September 1982.

0007-0920/83/010103–07 $01.00

### Materials and methods

### *Cytogenetics*

Cells were harvested for cytogenetic analysis using a modification of the technique of Trent & Salmon (1980). Briefly, CFUs were allowed to grow for 72–96 h in 7.5% $CO_2$. Colcemid (0.05 $\mu$g/ml) was added the last 4–12 h of culture. Clusters or colonies were then removed from the plates, washed free of methylcellulose with fresh medium, and placed into hypotonic (0.075 M KCl prewarmed to 37°C) for 25 min. Following centrifugation (150 × g, 5 min), the supernatant was removed and 5 ml of fresh cold fixative (3:1 methanol:glacial acetic acid) was added with vigorous vortex agitation. Cultures were then washed twice more with fixative and stored at −9°C. Air-dried slides were prepared and cells banded by G- (Sun *et al.*, 1973) or C- (Miller *et al.*, 1976) banding techniques. Chromosome harvesting of direct bone marrows was performed utilizing direct and short term liquid culture techniques (Morse *et al.*, 1977; Shiloh & Cohen, 1978).

All chromosome changes listed are "clonal" alterations according to ISCN (1978) definition.

### *Leukaemia cell culture*

Leukaemic cells were obtained from samples of bone marrow or peripheral blood and cultivated in methylcellulose as described by Buick *et al.* (1977). In brief, mononuclear cells obtained by Ficoll-

© The Macmillan Press Ltd.

Hypaque density centrifugation, were rosetted with neuraminidase treated sheep red blood cells (ERF-C) to remove rosette-forming T lymphocyte precursors from precursors of leukaemic blast cells to be cultured. Two hundred thousand cells/plate were set up in triplicate for each patient sample. Following a 1 h exposure to control media, cells were washed thoroughly and plated in 0.8% (v/v) methylcellulose in Alpha Medium supplemented with 10% fetal bovine serum and 20% (v/v) PHA-stimulated leukocyte conditioned medium (PHA-LCM) (Buick *et al.*, 1977). Cultures were then incubated at 37°C in a humidified atmosphere of 5% $CO_2$ with air for 7 days. Plates were examined for growth and categorized as no growth, minimal growth (e.g., cell doublings), clusters (<30 cells), or colonies (≧30 cells). Routine morphologic and histochemical analysis with Wright-Giemsa, Sudan Black B and chloracetate esterase staining was also used to confirm the leukaemic nature of colonies.

*Tritiated thymidine labelling of plated leukaemic cells*

Bone marrow (1–3 ml) was aspirated aseptically into a syringe containing sodium heparin. One-quarter volume of 3% dextran was then added to each sample in order to sediment red cells. The supernatant plasma (which was contained in the marrow cells) was transferred to a sterile centrifuge tube and diluted to 45 ml with Hank's Balanced Salt Solution with 10% fetal bovine serum (HBSS-FBS) and centrifuged 10 min at 600 x g. The cell button was then resuspended in HBSS-FBS, with the centrifugation and cell washing procedure repeated twice. A 2-ml aliquot of the washed cell suspension was placed in a sterile plastic tissue culture tube and warmed to 37°C in a waterbath. Five $\mu$Ci of [$^3$H]-dT (specific activity, 40–60 Ci/mM) was added to the cell suspension. After 1 h of incubation, the suspension was washed free of the unincorporated [$^3$H]-dT by 2 additional washes with HBSS-FBS. Following incubation, cell preparations were free of clumping and had a viability of ≧98%. Slides for autoradiography and microscopic examination were prepared utilizing a cytocentrifuge. Slides for autoradiography were then dipped in Kodak NTB-3 emulsion and further processed using high speed scintillation autoradiography (HSARG) (Durie & Salmon, 1975). Routinely, duplicate slides were exposed for 6 h and 24 h and without scintillator and compared with conventional autoradiography ([$^3$H]-dt, 18 Ci/mM exposure, 7 days, 2°C). No significant differences were found in final labelling indices (Durie & Salmon, 1975). Readings of slides processed using the HSARG technique and exposed for 24 h were used in this analysis.

Following developing of slides, each slide was stained with acid Giemsa stain. Autoradiographs prepared in this fashion were of high quality with a background median grain count of <35 grains/100 cells. Cells containing ≧5 grains over the nucleus were considered labelled. One thousand cells were counted in order to determine the LI, which was expresssed as a percentage. The median grain count of labelled leukaemic cells measured with this LI technique was ≧50 grains/cell nucleus. Statistical considerations regarding threshold grain counts and relation to background were as described by Clarkson *et al.* (1967).

*Patient population*

A total of 46 patients had bone marrow and/or peripheral blood samples referred to our laboratories for direct cytogenetic analysis and clonal assay. Of these, 7 had CML in the chronic or blastic phase, the remaining 39 having AML which was categorized using the FAB classification (Gralnick *et al.*, 1977). The patients in this study ranged in age from 12–74 years. Of 39 AML patients, 31 were classified as M1 or M2; 6 as M4; and one each M3 and M6. Eight patients were previously untreated with the remainder being evaluated at the time of first or later relapse. Drug treatment of these patients will be reported elsewhere (Durie *et al.*, submitted for publication).

### Results

We compared the success rate of obtaining analyzable mitoses from direct marrow cells and leukaemic CFUs. Forty-one of 46 (91.1%) of direct marrows and 30/36 (83.3%) methylcellulose cultures provided sufficient mitoses for cytogenetic analysis. This difference was not statistically different ($n=72$, $P=0.40$). As examples, comparison of the chromosome banding patterns of direct marrows and CFUs from 5 patients with AML and one patient with Ph$^1$ positive CML are described. One AML patient was studied twice over a 6-month period. Clinical and cytogenetic assessments of each of these patients are presented in Tables I and II.

Cytogenetic analysis of patients presenting with an abnormal karyotype revealed the presence of identical clonal chromosomal abnormalities in direct harvests and CFUs (Table 2, Figures 1–3). However, karyotypically normal mitoses (with or without E-rosette depletion prior to culture) were also observed in cultures from all 5 AML patients (Table II). We have found no consistent change in the ratio of normal:abnormal cells in direct *vs.*

**Table I** Clinical features of six patients and colony growth characteristics

| *Case No.* | *Sex* | *Age* | *Diagnosis* | *Disease status* | *Marrow or blood* | *E-RFC performed* | *Colony data Average # colonies/ $10^5$ cells* | *Days of culture*† | *Labelling index* |
|---|---|---|---|---|---|---|---|---|---|
| 1 | F | 74 | AML | 1st Relapse | Blood | No | 380 | 10 | 8% |
| 2 | M | 19 | AML | 3rd Relapse | Marrow | No | ND* | 10 | 19% |
| 3a | F | 21 | AML | 1st Relapse | Marrow | No | 160 | 7 | 4% |
| 3b | - | -- | --- | 2nd Relapse | Marrow | No | 100 | 6 | ND |
| 4 | M | 11 | AML | Relapse | Marrow | Yes | ND | 6 | ND |
| 5 | M | 72 | AML | 1st Relapse | Blood | Yes | 337 | 3 | ND |
| 6 | F | 65 | CML | Blastic crisis | Marrow | Yes | 1,050 | 7 | ND |

*ND = Not Determined.
†Day of culture of cytogenetic analysis.

**Table II** Summary of cytogenetic data from six patients

| *Case no.* | *Total banded cells analyzed (culture)* | *Abnormal karyotype* | *Percentage of cells with abnormal karyotype (culture)* |
|---|---|---|---|
| 1 | 15 (25) | — | — |
| 2 | 23 (50) | 45,XY,-14† | 60 (46) |
| 3a | 15 (12) | 45,X,+20,−21,t(8;16)(q22q24) | 33.3 (91.6) |
| 3b | 20 (33) | 45,X,+20,−21,t(8;16)(q22q24) | 15.0 (51.5) |
| 4 | 25 (46) | 45,XY,−7 | 94.1 (45.6) |
| 5 | 25 (25) | 46,XY,−12,+t(1;12)(q21p13) | 56.0 (91.4) |
| 6 | 20 (29) | 46,XX,t(9;22)(q34q11) | 100 (100) |

†The only clonal abnormality observed in this patient was −14. However, random chromosome loss+gain, and substantial chromosome breakage was also observed.

cultured cells. Both increased (cases 3a, 3b, 5) and decreased (cases 2, 4) percentages of abnormal cells were observed in cultured samples. Finally, we have been unable to recognize karyotypically unique clonal populations in cultured cells which were not found in our direct preparations.

One major advantage provided by the colony assay was the acquisition of a large number of mitoses suitable for chromosome-banding analysis. With one exception (case 3), chromosome-banding patterns of cultured cells were superior to direct preparations from our laboratory. For example, an unusual translocation was observed in direct and colony forming cells from case 5. G-banding of direct chromosones revealed a 46,XY karyotype with extra material translocated onto the short arm of chromosome 12, G-, and C-banding analysis from CFUs provided specific information on the donor

**Figure 1** Representative G-banded metaphase from LCFUs of case 5. Approximately 90% of all mitoses evidenced a single clonal karyotypic abnormality; the translocation of chromosomes 1 and 12 [t(1;12)(1qter→1q21::12p13→12qter)]. Arrow indicates the extra copy of chromosome 1q21→qter translocated onto the short arm (p13) of chromosome 12.

chromosome involved, and breakpoints of the translocation: t(1; 12)(1qter→1q21.:12p13→12qter) (Figures 1 and 2).

Characterization of AML colony-forming cells was also derived from examining the relationship between growth in methylcellulose, with LI and/or karyotype. Twenty-three patients were evaluated for LI and subsequently for colony growth. A highly significant correlation existed between a high LI ($\geqq 5\%$) and leukaemic colony formation ($P = 0.018$) (Table III). Similarly, when the karyotype of direct marrows was correlated for growth of leukemic CFUs, a high correlation existed between the presence of any karyotypically abnormal clone and colony growth ($P = 0.010$) (Table III). When the LI and karyotype for samples with or without leukaemic colonies were cross-correlated, no significant difference was observed ($P = 0.80$; 0.20, respectively). However, this apparent lack of correlation is explained when leukaemic colony growth and LI (%) were compared from chromosomally normal and abnormal cases. A highly significant correlation existed between growth and high LI for chromosomally abnormal samples ($P = 0.018$), while chromosomally normal samples did not demonstrate a significant correlation ($P = 0.28$) (Table III).

Because of the possibility of significant differences between bone marrow and blood CFUs from the same patient, results from marrow and peripheral blood CFUs were compared. In 4 patients, cytogenetics and LI could be compared from both blood and marrow samples. Karyotype (normal or abnormal) and LI did not differ between sample sites in any of these patients. One additional patient had CFUs from the peripheral blood only and therefore could not be compared. The proportion of cases studied for karyotype or LI versus growth in this assay was similar with or without samples derived from peripheral blood.

**Figure 2** Representative C-banded metaphase from LCFUs of case 5. Further confirmation of the involvement of chromosome 1q is revealed by the presence of a large block of constitutive heterochromatin (arrow) on the translocation chromosome.

**Figure 3** G-banded metaphase from LCFUs of a patient with CML (case 6). All cells evidenced the Ph[1] translocation between chromosomes 9 and 22 [t(q;22)(q34q11)]. The Ph[1] chromosome was the only clonal change noted in both direct and cultured cells from this patient.

**Table III** Interrelationship between leukaemic colony growth, direct karyotype, and tritiated thymidine labelling index

| | *Leukaemic colony growth (35 pts; 49 studies)* | *Direct karyotype† (23 pts; 49 studies)* | | *Labelling index (LI)† (28 pts; 31 studies)* | | | |
|---|---|---|---|---|---|---|---|
| | | | | *Normal karyotype* | | *Abnormal karyotype§* | |
| | | *Normal* | *Abnormal* | *LI–5%* | *≧5%* | *LI–<5%* | *≧5%* |
| Growth | | | | | | | |
| No. studies | 32/49 | 13/27 | 19/22 | 1/5 | 7/11 | 1/4 | 11/11 |
| Percent | (65%) | (48%) | (86%) | (20%) | (64%) | (25%) | (100%) |

*The Fisher exact test and log-linear model methods were used to statistically compare the indicated percentages.

†Growth more likely with abnormal karyotype: $p = 0.010$*

‡Growth more likely with labelling index (≧5%): $p = 0.018$.

§Growth most likely with *both* abnormal karyotype and high labelling index (≧5%): $p = 0.018$.

### Discussion

The success rate in our laboratory of obtaining analyzable mitoses from leukemic CFUs was similar to that observed in our laboratory for direct harvesting techniques. However, the requirement of greatly increased sample preparation, coupled with the time-consuming nature of cytogenetic analysis, would appear to make utilization of this colony assay for routine cytogenetic analysis too laborious. Also, in contrast to recent reports utilizing liquid culture (Knuutila *et al.*, 1981), we have been unable to recognize clonal subpopulations in CML or AML unique to or with increased frequency in colony-forming cells. Thus, although the mitotic index of samples was often increased from CFUs, identification of clonal subpopulations was not increased. However, the colony assay was very useful in providing large numbers of mitotic cells suitable for detailed chromosome banding analysis.

Cytogenetic examination of CML in blast crisis (case 6) revealed only the presence of $Ph^1$ positive cells (Figure 3). With one exception, (Sonoda *et al.*, 1980), these results are similar to previous reports of CFUs from patients with CML (Chervenick *et al.*, 1971; Moore & Metcalf, 1973). Of perhaps more interest was the consistent finding in AML patients of karyotypically "normal" cells from cultures also displaying colonies with an abnormal karyotype (Table II, cases 2–5). Although we cannot exclude the possibility of non-leukaemic CFUs growing in this system, it is possible that these karyotypically "normal" mitoses may, in fact, have been of leukaemic origin and harbor subtle chromosomal rearrangements unrecognized with current techniques. Utilizing prometaphase chromosomes, Yunis *et al.* (1981) has recently suggested that 100% of acute non-lymphocytic leukaemia patients display a cytogenetic abnormality. We are currently adapting this methodology to colony cells to determine whether an increased percentage of CFUs with chromosomal abnormalities can be identified. Additionally, we are beginning cytogenetic analysis of single colonies which may provide further information on the nature of CFU growth in this assay.

Of major interest is our finding in AML patients that karyotypically abnormal and/or high LI had the highest percentage of growth in this blast cell assay (Table III). These results support previous findings from this laboratory on multiple myeloma (Durie *et al.*, in press), suggesting that the clonogenic fraction is largely derived from cycling marrow cells. These results may also explain the relationship of normal to leukaemic cell growth in agar culture observed in other assays (Verma *et al.*, 1979; Spitzer *et al.*, 1979). Verma *et al.* (1979) suggested that PHA-stimulated patients with only normal CFU-C growth (therefore negative for leukaemic growth) in agar culture display a prognostic advantage over patients with a high fraction of leukaemic colonies. Although we have not yet directly correlated our results on colony formation and survival, it is well known that the presence of karyotypically abnormal clones (Shiraishi *et al.*, 1982) or high labelling index (Durie, 1982) are indicators of poor patient prognosis. It is possible that our low LI-karyotypically normal population is similar to the category I-A (good prognosis) patients reported by Spitzer *et al.* (1979).

We feel that the cytogenetic and cytokinetic evidence presented in this report provides strong support for the leukaemic nature of CFUs grown in the clonal assay of Buick *et al.* (1977). This assay may provide a valuable means of providing a large number of leukaemic metaphases for detailed cytogenetic analysis.

We gratefully acknowledge the technical assistance of L. Norris-Kloos and S. Olson. We also thank Dr. T. Moon for assistance with statistical design. Dr. Trent is supported in part by Public Health Service grants CA-29476 and CA-17094 awarded by the National Cancer Institute, Department of Health and Human Services. Professor Durie is a scholar of the Leukaemia Society of America.

**References**

BUICK, R.N., TILL, J.E. & McCULLOCH, E.A. (1977) A colony assay for proliferative blast cells circulating in myeloblastic leukaemia, *Lancet*, **i**, 862.

CHERVENICK, P.A., ELLIS, L.D., PAN, S.R. & LAWSON, A.L. (1971) Human leukemic cells: in vitro growth of colonies containing the Philadelphia ($Ph^1$) chromosome. *Science*, **174**, 1134.

CLARKSON, B., OHKITA, T., OTAK, B. & FRIED, J. (1967) Studies of cellular proliferation in human leukemia. I. Estimation of growth rates of leukemic and normal hematopoietic cells in two adults with acute leukemia given single injections of tritiated thymidine. *J. Clin. Invest.*, **46**, 506.

DUBÉ, I.D., EAVES, C.J., KALAUSEK, D.K., & EAVES, A.C. (1981). A method for obtaining high quality chromosome preparations from single hematopoietic colonies on a routine basis. *Cancer Genet. Cytogenet.*, **4**, 157.

DURIE, B.G.M. (1982). Staging and kinetics of multiple myeloma. *Clin. Haematol*, **11**, 3.

DURIE, B.G.M., BROOKS, R.J., KLOOS, L., SEROKMAN, R., & MOON, T.E. (1982a). *In vitro* drug sensitivity testing of leukemic clonogenic cells. (Submitted for publication).

DURIE, B.G.M., SALMON, S.E., & YOUNG, L.A. (1982b). Kinetics of myeloma stem cell culture: correlations with in vitro and in vivo drug sensitivity. *Blood*, (In press).

DURIE, B.G.M. & SALMON, S.E. (1975). High speed scintillation autoradiography. *Science*, **190**, 1093.

GRALNICK, H.R., GALTON, D.A.G., CATOVSKY, D., SULTAN, C. & BENNETT, J. (1977). Classification of acute leukemia. *Ann. Int. Med.*, **87**, 740.

ISCN (1978). An International System for Human Cytogenetic Nomenclature. *Cytogenet. Cell Genet.*, **21**, 313.

KNUUTILA, S., VIROPIO, P., ELANEN, E. & 4 others (1981). Culture of bone marrow reveals more cells with chromosomal abnormalities than the direct method in patients with hematologic disorders. *Blood*, **58**, 369.

LÖWENBERG, B., SWAIT, K. & HAGEMEIJER, A. (1980). PHA-induced colony formation in acute non-lymphocytic and chronic myeloid leukemia. *Leukemia Res.*, **4**, 143.

METCALF, D. (1977). Hematopoietic Colonies: In Vitro *Cloning of Normal and Leukemic Cells.* New York: Springer-Verlag.

MILLER, D., TANTRAVAKI, T., DEV, V. & MILLER, O.J. (1976). Q and C-band chromosome markers in inbred strains of *Mus musculus*. *Genetics*, **84**, 67.

MOORE, M.A.S. & METCALF, D. (1973). Cytogenetic analysis of human acute and chronic myeloid leukemia cells cloned in agar culture. *Int. J. Cancer*, **11**, 143.

MORSE, H.G., HUMBERT, J., HUTTER, J.J. & ROBINSON, A. (1977). Karyotyping of bone marrow cells in hematologic diseases. *Human Genet.*, **34**, 33.

SHILOH, Y, & COHEN, M. (1978). An improved technique of preparing bone marrow specimens for cytogenetic analysis. *In Vitro*, **14**, 510.

SHIRAISHI, Y., TOYUCH, H., NIIYA, F., KIKUKAWA, K., KUBONISKI, S., OHMURA, T., AMAWAKI, M.L. & UEDA, N. (1982). Diagnostic and prognostic significance of chromosome abnormalities in marrow and mitogen response of lymphocytes of acute non-lymphocytic leukemia. *Cancer Genet. Cytogenet.*, **5**, 1.

SONODA, Y., IDE, T., MISAWA, S., ABE, T., TAKIRO, T. & KOHSAKI, M. (1980). Cytogenetic studies of granulopoietic colonies in patients with chronic myelocytic leukemia. *Acta Haematol. Jpn.*, **43**, 55.

SPITZER, G., VERMA, D., DICKE, K., SMITH, T. & McCREDIE, B. (1979). Subgroups of oligoleukemia as identified by in vitro agar culture. *Leukemia Res.*, **3**, 29.

SUN, N., CHU, E. & CHANG, C. (1973). Staining method for the banding patterns of human mitotic chromosomes, *Mamm. Chrom. Newsl.*, **14**,26.

TRENT, J.M. & SALMON, S.E. (1980). Human tumour karyology: marked analytic improvement via short term agar culture. *Br. J. Cancer*, **41**, 867.

VERMA, D.S., SPITZER, G., DICKE, K.A. & McCREDIE, B. (1979). *In vitro* agar culture patterns in preleukemia and their clinical significance. *Leukemia Res.*, **3**, 41.

YUNIS, J.J., BLOOMFIELD, C.D. & ENSRUD, K. (1981). All patients with acute non-lymphocytic leukemia may have a chromosomal defect. *N. Engl. J. Med.*, **305**, 135.